# Structural Characterization and Analysis of High-Strength Laminated Composites from Recycled Newspaper and HDPE

**DOI:** 10.3390/polym11081311

**Published:** 2019-08-06

**Authors:** Binwei Zheng, Chuanshuang Hu, Litao Guan, Jin Gu, Huizhang Guo, Weiwei Zhang

**Affiliations:** 1 College of Materials and Energy, South China Agricultural University, Guangzhou 510642, China; 2 Wood Materials Science, Institute for Building Materials, ETH Zurich, Stefano-Franscini-Platz, 38093 Zurich, Switzerland; 3 Guangxi Key Laboratory of Clean Pulp & Papermaking and Pollution Control, Nanning 530004, China

**Keywords:** recycled newspaper, laminated composites, density, porosity, microstructure

## Abstract

Recycled newspaper (NP) shows excellent potential as a reinforcement for polymer composites. Herein, high-strength laminated composites were prepared by using NP laminas as reinforcement and high-density polyethylene (HDPE) films as matrix. Physical and mechanical properties of the laminated composites were measured. It was found that the flexural strength of the composites had a good linear relationship to its density, with R^2^ = 0.9853. The flexural and tensile strength of the composites at the maximum density (1.40 g/cm^3^) reached up to 95.6 ± 2.4 MPa and 99.4 ± 0.8 MPa, respectively. SEM results showed that NP layer inside the composite became compact at the hot pressing time of 40 min, because the melted HDPE permeated into the NP layers to bond the NP fibers. Quantitative description of the composite porosity was conducted according to the density of the composite. The 24-h water absorption of the composite was highly related to its porosity, with R^2^ = 0.8994. This study reveals that density of laminated composites is an important parameter, which could be used to forecast the mechanical strength, and its derived value, porosity of the composites, could be used to predict the water absorption behavior of the composite.

## 1. Introduction

Efficient waste paper recycling plays an important role in the sustainable environment. We will benefit from reduction of landfill, saving energy, improving organization image, diverting materials from disposal, conserving natural resources, and reducing greenhouse gas emissions through waste paper recycling [1]. The recycling of waste paper is improvable worldwide. China consumed 104.2 million tons of paper and board in 2016; meanwhile, only 49.6 million tons were recovered for paper products. Nevertheless, additional cleaning processes are needed to remove contaminants such as ink particles, inorganic fillers, and coatings in recycled paper. Subsequent wastewater and sludge treatment increase the production cost. Furthermore, the fiber of waste recycled paper is only reusable for a maximum of seven times because of the degradation of the fiber quality [2].

Novel recycled paper fiber-based products have been explored and developed via either chemical or physical routes during the last decades. Some high-value cellulosic products have been successfully prepared from recycled paper, such as carboxymethyl cellulose (CMC) [3], cellulose nanofibers (CNFs) [4], and cellulose nanocrystals (CNCs) [5] due to its advantages of high content of cellulose but low content of lignin. Recycled paper fiber has also been used to produce biogas [6], ethanol [7], and compost [8].

Recycled paper fibers have great potential for polymer composites as a reinforcement because they are the millimeter-scale length and micro-scale diameter with robust mechanical properties. Recently, recycled paper fiber as reinforcement for polymer composites has been reported [9,10,11,12,13,14]. Interface modification using maleated type coupling agent and mechanical properties of polypropylene composites reinforced with old newspaper, kraft pulp, and hemp were studied [14]. Chopped glass fiber and recycled newspaper cellulose fiber-reinforced polylactic acid composites were developed using a full-size twin-screw extruder and an injection machine [12]. Compatibilization and the structure–properties relationship of high-density polypropylene-based composites using recycled multilayer cartons as cellulose source were proposed [9]. Lignocellulosic fibers from the newspaper were modified with acetic anhydride, NaOH, and KMnO_4_ to enhance the composite interfacial adhesion between polyvinyl chloride matrix and the newspaper fibers and to avoid the inherent susceptibility of natural fibers to moisture expansion [13]. In the above studies, paper fibers are commonly mixed with polymer matrices using twin-screw extruder and injection molding. It was reported that the fiber length decreased from 0.654 mm before compounding to 0.089 mm after injection molding, corresponding length to diameter ratio from 65 to 18 [15]. The fiber aspect ratio reduction resulted in more fibers pulling out of the matrix rather than fracture when the interfacial bonding was unchanged, meaning that the reinforcing effect was greatly reduced.

Polymer laminated paper composites could effectively avoid the fiber length reduction and thus displayed high mechanical properties [16,17,18,19,20]. Paper fibers were prefabricated into mats, which were subsequently stacked with thermoplastic polymer films or impregnated with a liquid matrix. The stack was hot pressed into laminated composites. This preparation process not only avoided the damage to paper fibers but also obtained a composite structure where the fiber orientation factor increased from 1/5 to 3/8 [21]. Paper hand-laminas impregnated with resin were used to prepare pulp fiber-reinforced unsaturated polyester composites, which had tensile moduli and tensile strength comparable with those of glass fiber-reinforced composites [16]. The maximum composite tensile strength was up to 121 MPa when combing wet-laid paper-making process with polylactic acid film stacking process [21]. Three different kinds of papers were adopted as reinforcements to produce laminated composites, and the results showed that the laminated composite with up to 30 vol.% newspaper (NP) had a tensile and flexural strength of 58 MPa and 68 MPa, respectively [19]. The addition of 5 wt % MAPP could increase the tensile properties of the laminated composites by 10%–20% [20].

In this study, high-strength laminated composites were prepared using recycled NP sheet as a reinforcement and HDPE films as matrix. Effects of the porosity on physical and mechanical properties of the laminated composites were explored. Microstructure was observed to investigate the reinforcing mechanism of NP laminas.

## 2. Materials and Methods

### 2.1. Materials

High-density polyethylene (HDPE; density 0.95 g/cm^3^; MFR 0.24 g/10 min at 190 °C/2.16 kg; T_m_ 119 °C) films with the measured thickness of 12.8 ± 0.3 μm, were purchased from Charoen Pokphand Group, Guangzhou, China. Newspaper (NP) was recycled from Nanfang Metropolis Daily, China. The main components contained 46.9% cellulose, 24.0% hemicellulose, 16.5% lignin, and 10.9% ash. Tensile strength of the NP was measured to be 35.8 MPa and reached up to 81.5 MPa after 40 min hot pressing at 160 °C according to ASTM D828-16 [22].

The NP sheet was cut into 5 cm × 5 cm to measure its mass and thickness (average value: 49.4 μm). The apparent density of the NP sheet (ρ*_NP_*) was calculated by the mass (*m_NP_*) divided by the volume (*V_NP_*). Its average value was 0.89 g/cm^3^. The porosity of the NP sheet (*P_NP_*) was determined according to microporous functional membrane measurement for porosity according to GB/T 33052-2016 [23]. Toluene was selected as absorption liquid because cellulosic fibers could be wetted but not swelled up by toluene. The volume of pores inside the NP sheet was equivalent to the volume of toluene inside the NP sheet. *P_NP_* was measured to be 55.9% according to Equation (1).

(1) $$P_{NP}\%=\frac{\frac{m_{a}-m_{NP}}{\rho_{t}}}{V_{NP}}\times 100\%$$

where *m_NP_* is the mass of the NP sheet; *m_a_* is the mass of the NP sheet after absorption of toluene; *ρ_t_* is the density of toluene; *V_NP_* is the volume of the NP sheet.

The volume of the NP sheet including the volume of pores and the volume of NP fibers can be described according to Equation (2). Therefore, the fiber density (ρ*_f_*) of the NP sheet was calculated to be 2.02 g/cm^3^. The value was larger than pure fiber density (1.55 g/cm^3^), which was reasonable because some high-density additives of calcium carbonate and kaolin existed in the NP sheet and the calculated value (ρ*_f_*) in Equation (2) was the density of mixed solids instead of pure cellulosic fibers.

(2) $$V_{NP}=\frac{m_{a}-m_{NP}}{\rho_{t}}+\frac{m_{NP}}{\rho_{f}}$$

### 2.2. Composite Fabrication

**Figure 1:**
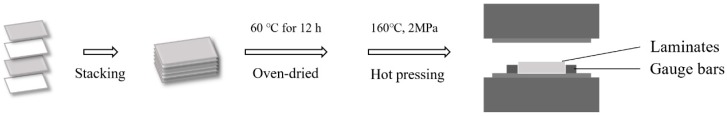
Schematic diagram of newspaper (NP)/high-density polyethylene (HDPE) laminated composites fabrication.

The mass ratio was adopted to investigate the influence of recycled NP content on properties of HDPE laminated composites. Each NP sheet was stacked with one, two, or three layers of the HDPE film, which were corresponding to the laminated composite containing 78.5 wt %, 64.6 wt %, or 54.9 wt % paper content, respectively. The stack with the dimension of 27 cm × 38 cm was oven-dried at 60 °C for 12 h to keep the water content of the NP sheet constant. The pre-dried stack was then transferred to the pre-heated hot press machine (BY302X2/2 150T, Suzhou New Cooperative, Suzhou, China) at 160 °C. Two 5-mm thick gauge bars were placed firmly beside the two longitudinal edges of the stack to ensure the final thickness of the laminated composite. The pressing pressure and the heating temperature were set to be 2.0 MPa and 160 °C, respectively. The different hot pressing time durations of 20, 30, 40, and 50 min were conducted to study the influence of the heating time duration on the properties of the laminated composite after the stack was input the chamber. It took about 20 min for the middle layer to reach a steady temperature. The heating panel was quickly cooled down to 60 °C using circulation water cooling system after the hot-pressing procedure was completed. Finally, the pressure was released, and the laminated composite was removed. The fabrication of newspaper (NP)/high-density polyethylene (HDPE) laminated composites are depicted in Figure 1.

The apparent density of the laminated composite (ρ*_c_*), which is defined as the mass divided by the volume (*V_c_*), was calculated through weighting 5 cm × 5 cm sample and measuring the thickness to calculate its volume.

### 2.3. Composite Morphology

The laminated composite was cut into bars with the cross-sectional dimensions of 10 mm (length) × 5 mm (thickness). To analyze the microstructure of the NP/HDPE laminated composites, bars were soaked in liquid nitrogen for 20 min before they were cut along the cross-section, and the cross-section cutting surfaces were cleaned by a microtome (SM2000R, Leica, Shanghai, China). The bars were then mounted onto metal substrates and coated with carbon thin film for conductivity. The morphology of composite cross-section was observed by a scanning electron microscopy (EVO-18 BSEM, Zeiss, Jena, Germany) using an accelerating voltage of 30 kV.

### 2.4. Composite Properties

The tensile and flexural properties of the laminated composites were measured using an electromechanical universal testing machine (CMT5504, Shenzhen Rethink Cooperation, Shenzhen China) according to GB/T 1040-2006 [24] and GB/T 9341-2008 [25], respectively. Dumbbell specimen was used for measuring the tensile strength and the loading speed was 5 mm/min. The flexural specimen with the dimensions of 250 mm (length) × 50 mm (width) × 5 mm (thickness) was used for measuring the flexural strength and flexural modulus. The loading speed was 2.7 mm/min. Five replicates were tested to obtain the average values of the mechanical properties and their standard deviations.

The water resistance test was conducted according to GB/T 1034-2008 [26]. All the specimens with the dimensions of 50 mm (length) × 50 mm (width) × 5 mm (thickness) were oven-dried at 50 ± 2 °C for 24 h before water soaking and then immersed in distilled water for 24 h at 23 ± 2 °C. Each specimen was weighed and measured for thickness at the three marked locations. Five replicates were tested.

## 3. Results and Discussion

### 3.1. Physical and Mechanical Properties of the Laminated Composite

**Figure 2:**
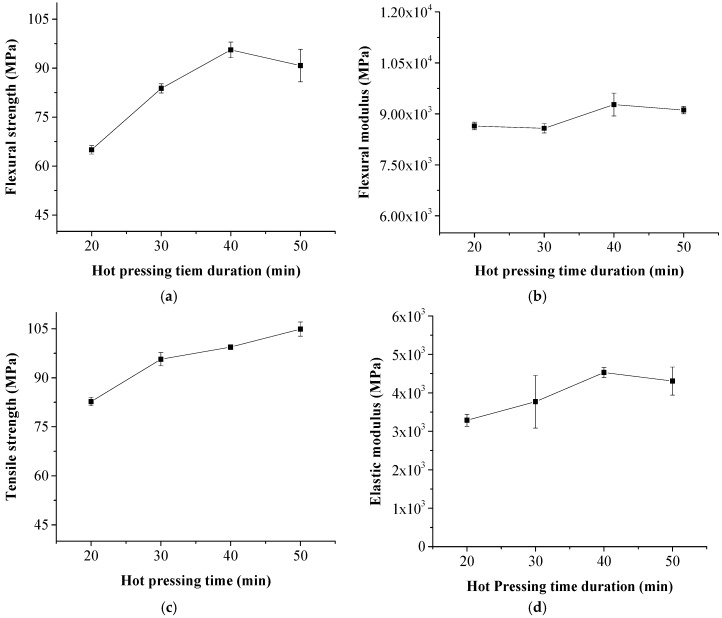
Effects of the hot pressing time duration on mechanical properties of the composite. (**a**) flexural strength; (**b**) flexural modulus; (**c**) tensile strength; (**d**) elastic modulus.

HDPE films melted and penetrated into the pores of NP sheet to form a compact structure during the hot-pressing; the NP sheet/HDPE films stack to the laminated composite at high temperature. Effects of the hot pressing time duration on mechanical and physical properties of the laminated composite are shown in Figure 2. The flexural and tensile strengths increased from 82.7 MPa to 99.4 MPa, and 65.0 MPa to 95.6 MPa; meanwhile, the flexural and tensile modulus increased from 8500 MPa to 9500 MPa, and 3286 MPa to 4530 MPa when the hot pressing time duration increased from 20 min to 40 min with 64.6 wt % paper content. The flexural strength slightly decreased when the hot pressing time exceeded 40 min. However, the tensile strength continued to increase with the hot pressing time of 50 min. The mechanical strengths of the laminated composite in this study were comparable to the mechanical properties of the composites reinforced with glass fibers or even carbon fiber [27,28,29] and much greater than those of injection molding paper-fiber composites [30]. The high mechanical strengths were obtained because the paper fiber content was higher than 64.6 wt % and paper fiber aspect ratio was not mechanically reduced in the laminated composite. The paper fiber content in injection molding paper–fiber composites was usually less than 50% because of the limits of the paper fiber injecting friction [21,31].

**Figure 3:**
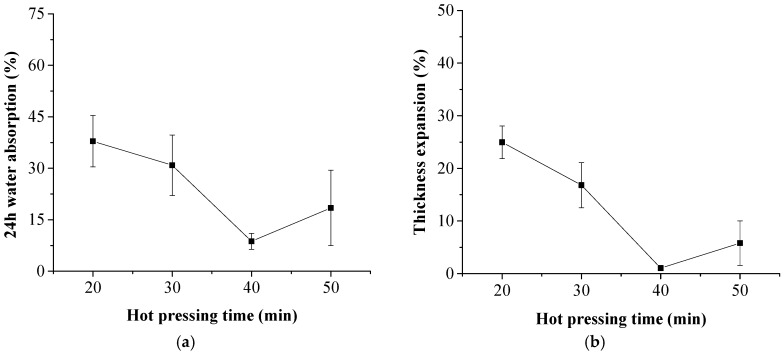
Effects of the hot pressing time duration on water absorption and thickness expansion of the composite. (**a**) 24-h water absorption; (**b**) thickness expansion.

Effects of the hot pressing time duration on the water absorption behavior of the laminated composites are shown in Figure 3. The 24-h water absorption and the thickness expansion of the composite decreased from 37.9% to 8.7% and 25.0% to 1.1% with the increase of the hot pressing time duration from 20 min to 40 min. Pores and voids in the NP layers provided a pathway for water transmission due to the capillary effect. The pathway was partially blocked with the permeation of the melted HDPE into NP layers during hot pressing procedure. The 24-h water absorption and the thickness expansion rebounded slightly when the hot-pressing time exceeded 40 min.

**Figure 4:**
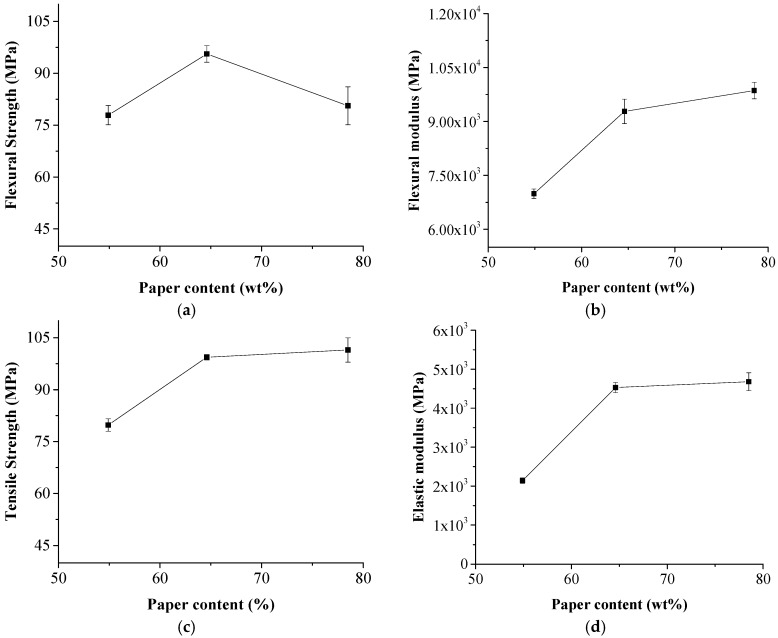
Effects of NP content on mechanical properties of the composite. (**a**) flexural strength; (**b**) flexural modulus; (**c**) tensile strength; (**d**) elastic modulus.

To study the effects of the NP content on the physical and mechanical properties of the laminated composite, the hot pressing time duration was set to be 40 min. The thickness of the purchased HDPE film was 12.8 μm so that the NP content of the composite was controlled by stacking each piece of the NP laminas with one, two, or three layers of HDPE films, which corresponded to NP contents of 78.5 wt %, 64.6 wt %, and 54.9 wt %, respectively. Effects of the NP content on the mechanical properties of the composite are shown in Figure 4. The flexural strength of the composite reached the maximum 95.6 MPa when the NP content was 64.6 wt %. The excessive high content of the NP laminas had a negative effect on the flexural strength. The tensile strength of the composite continued to increase to 101.5 ± 3.5 MPa when paper content increased from 64.6 wt % to 78.5 wt %. The flexural modulus and the elastic modulus increased from 6990 ± 130 MPa to 9860 ± 230 MPa, and 2140 ± 70 MPa to 4680 ± 230 MPa when paper content increased from 54.9% to 78.5%. It was thought that modulus of the composite was mainly related to the reinforcement content while the mechanical properties were affected by not only the NP reinforcement, but also the interface bonding between the NP layers and the HDPE layers.

**Figure 5:**
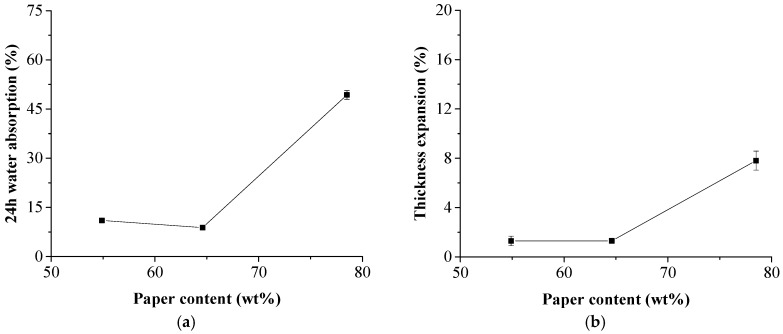
Effects of NP content on water absorption and thickness expansion of the composites. (**a**) 24-h water absorption; (**b**) thickness expansion.

Effects of the NP content on 24-h water absorption and the thickness expansion are shown in Figure 5. The results showed that the water absorption behavior of the composite was similar when paper content was between 54.9 wt % and 64.6 wt %. However, there was a sudden increase of water absorption and thickness expansion when the paper content was 78.5%, which may be due to a lack of melt HDPE polymers to block the pores and voids of the NP laminas.

**Figure 6:**
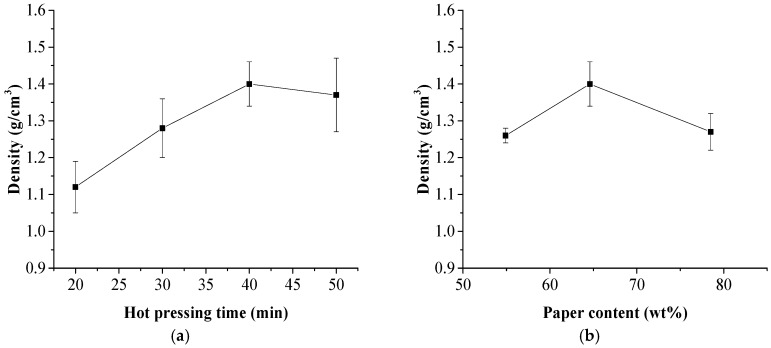
Effects of hot pressing time duration and NP content on the density of the laminated composite. (**a**) hot pressing time; (**b**) paper content.

Effects of hot pressing time duration and the NP content on the densities of the laminated composites are shown in Figure 6. The density of the laminated composite increased from 1.12 g/cm^3^ to 1.4 g/cm^3^ with the increase of the hot-pressing time duration from 20 to 40 min. It was thought that more compact structure was obtained because the NP laminas were compressed under the pressing pressure and the melt HDPE penetrated into pores and voids of the NP layers. The maximum density of the composite was obtained when the NP content was 64.6 wt %. The density of the composite was affected by the coupling effect of the composite components and their interaction. The melt HDPE film could perfectly permeate and fill the pores and voids of the NP laminas because the 64.6 wt % NP content may be the optimum formula.

**Figure 7:**
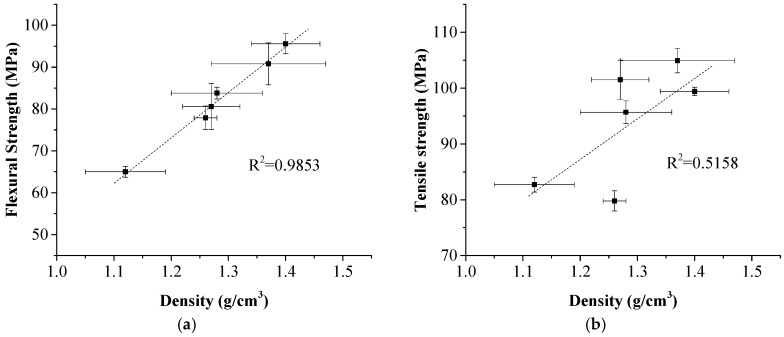
Relationship between density and mechanical strength of the laminated composites. (**a**) flexural strength; (**b**) tensile strength.

Statistical analysis results of the density and mechanical strength of the composite are shown in Figure 7. It can be observed that a good linear relationship existed between the flexural strength and the density with a correlation coefficient R^2^ of 0.9853 and between the tensile strength and the density with a correlation coefficient R^2^ of 0.5158. This finding was meaningful and valuable because the flexural and tensile strengths of the laminated composites could be quickly predicted through measuring its density.

### 3.2. Microstructure of the Laminated Composite

**Figure 8:**
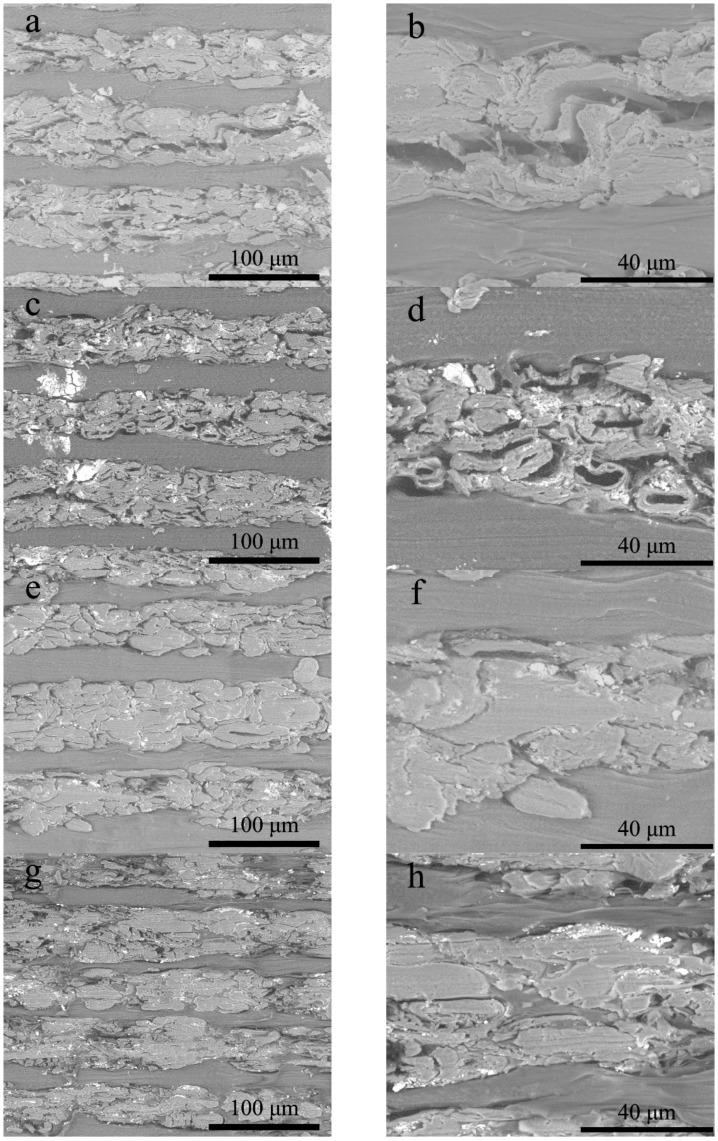
Morphological images of the composite cross sections at different hot pressing time. (**a**,**b**) 20 min; (**c**,**d**) 30 min; (**e**,**f**) 40 min; (**g**,**h**) 50 min.

The morphology of the composite cross section obtained by BSEM is shown in Figure 8. It was observed that there were some white dots scattering on the cross-section of the NP laminar, which was regarded to be inorganic fillers of calcium carbonate and kaolin. A lamellar structure of NP and HDPE layers was clear. The interfacial bond between NP and HDPE layers was due to the mechanical interlocking rather than the chemical binding since no coupling agents were added. The NP layers in the composites prepared at hot pressing time durations of 20 and 30 min was not well compacted, and pores and voids were obviously observed in Figure 8b,d. With the increase of hot-pressing time duration to 40 min, the majority of pores among the fibers disappeared because the NP layers became compact. It was conjectured that a small amount of HDPE had penetrated into the NP layer and functioned as an adhesive so that fibers were bonded tightly to form a dense structure. The variation of micro-/macro-porous structure in the laminated composites well explained its densification with the increase of hot pressing time. The dense structure of the laminated composites was favorable to improve the mechanical strength because the enhanced friction among fibers increased the difficulty of fibrous pulling out [32]. Although the recycled NP laminas was a short-fiber cross-linked mat, its reinforcing effect could be comparable to these laminated composites prepared with long-fiber mat [33,34] when the NP fibers were tightly bonded in compact state. The mechanical strength of NP layers inside the laminated composite was variable with the degree of structural compactness.

It can be observed from Figure 8g,f that visible HDPE occupied certain space inside NP layers and the NP layers lost its integrity and continuity, which resulted in a slight fluctuation in the density of the laminated composite. The breakage of the NP layer as a reinforcement might reduce the mechanical strength of the laminated composite. Note that there was still a few voids that existed in the NP layers as seen in Figure 8f,h. When the hot-pressing time was over 50 min, excess HDPE penetrated into the NP layers to break its continuity and integrity.

The pores and voids of the NP laminar provided the pathway and space for water absorption. Therefore, quantification of the pores and voids in the NP layer was necessary. After hot pressing and cooling solidification, NP layer inside the composite was compressed and part pores and voids were occupied by melt HDPE. Herein, the volume of composite included three parts: NP fibers, HDPE matrix, and residual pores. The density NP fibers (ρ*_f_*) was 2.02 g/cm^3^ as previously calculated. The volume of residual pores (*V_p_*) and the porosity of composite (*P_c_%*) was calculated as Equations (3) and (4), as follows:

(3) $$V_{p}=\frac{m_{c}}{\rho_{c}}-\frac{m_{NP}}{\rho_{f}}-\frac{m_{HDPE}}{\rho_{HDPE}}$$

(4) $$P_{c}\%=\frac{V_{p}}{V_{c}}\times 100\%.$$

**Figure 9:**
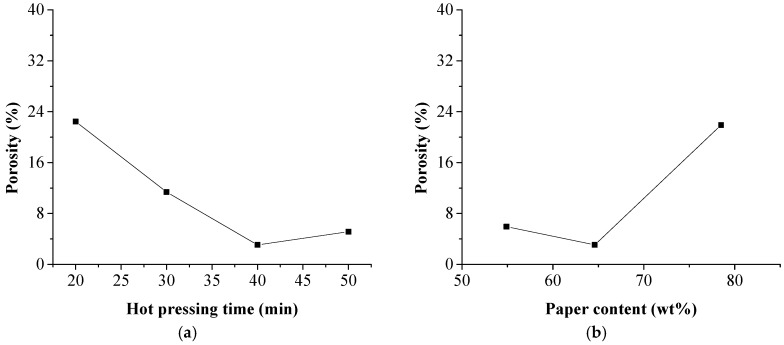
Effect of hot pressing time and NP content on porosity of the laminated composites. (**a**) hot pressing time; (**b**) paper content.

Effects of the hot-pressing time duration and paper content on the porosity of the composites are shown in Figure 9. A gradual increase of the composite density meant a gradual decrease in porosity of the laminated composites. The lowest porosity value was calculated to be 3.06%, corresponding to the sample with the highest density of 1.4 g/cm^3^. The small amount of void in NP layers could be confirmed in Figure 8f. The high porosity in the sample prepared with hot-pressing time duration of 20 min was due to incomplete permeation of HDPE while the sample with 78.5 wt % paper content probably contributed to deficient HDPE to bond the NP fibers.

**Figure 10:**
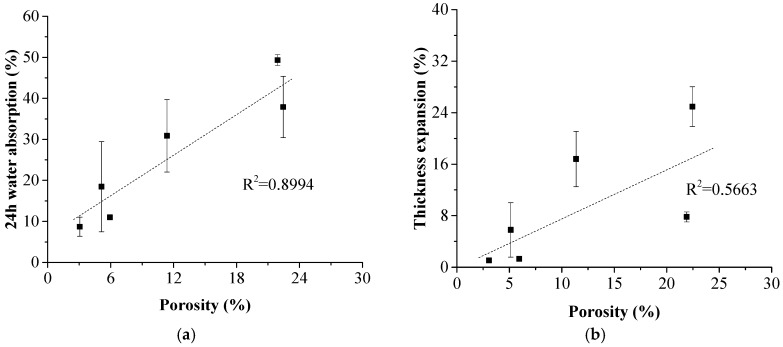
Relationship between porosity and water absorption of the laminated composites. (**a**) 24-h water absorption; (**b**) thickness expansion.

The relationship of the porosity and water uptake behavior of the laminated composites is shown in Figure 10. Since pores and voids inside the hydrophilic NP layers provided the pathway and space for water uptake, the water absorption was highly related to the porosity of the composite with R^2^ = 0.8994. However, the relationship between thickness expansion and porosity was not obvious. The thickness expansion of the composites was influenced not only by the water absorption capacity, but also the internal binding force inside the NP layer. For the sample prepared with hot-pressing time duration of 20 min, incomplete permeation of HDPE resulted in low internal binding force in the NP layers and the thickness expansion was up to 24.95 ± 3.08%. However, HDPE had already penetrated into the NP laminas to bond the NP fibers in the sample with 78.5 wt % paper content. Although the amount of HDPE was insufficient, the internal binding force produce by the HDPE was much higher to control the thickness expansion in a low level, only 7.81 ± 0.77%.

## 4. Conclusions

A high-strength laminated composite was developed using recycled NP sheet and HDPE films. During the hot pressing process, the melted HDPE penetrated into the NP layers to form a compact structure and excessive infiltration led to destruction of NP layers. The physical and mechanical properties of the laminated composite mainly depended on the degree of NP layer compactness and its integrity. There was a good linear relationship between the flexural strength and the density. The maximum density and flexural strength were 1.40 g/cm^3^ and 99.4 ± 0.8 MPa, respectively. The water absorption behavior was highly related to the porosity of the composite, since pores and voids inside the hydrophilic NP layers provided the pathway and space for water uptake. In comparison, the thickness expansion of the composites was influenced by both the water absorption capacity and the internal binding force. The density was proved to be an important parameter to predict the mechanical and physical properties of the laminated composites.
